# Identification of a Specific Plasma Sphingolipid Profile in a Group of Normal-Weight and Obese Subjects: A Novel Approach for a “Biochemical” Diagnosis of Metabolic Syndrome?

**DOI:** 10.3390/ijms24087451

**Published:** 2023-04-18

**Authors:** Antonello E. Rigamonti, Michele Dei Cas, Diana Caroli, Alessandra De Col, Silvano G. Cella, Rita Paroni, Alessandro Sartorio

**Affiliations:** 1 Department of Clinical Sciences and Community Health, University of Milan, 20129 Milan, Italy; silvano.cella@unimi.it; 2 Department of Health Sciences, University of Milan, 20142 Milan, Italy; michele.deicas@unimi.it (M.D.C.); rita.paroni@unimi.it (R.P.); 3 Istituto Auxologico Italiano, Istituto di Ricovero e Cura a Carattere Scientifico (IRCCS), Experimental Laboratory for Auxo-Endocrinological Research, 28824 Piancavallo-Verbania, Italy; d.caroli@auxologico.it (D.C.); a.decol@auxologico.it (A.D.C.); sartorio@auxologico.it (A.S.); 4 Istituto Auxologico Italiano, Istituto di Ricovero e Cura a Carattere Scientifico (IRCCS), Experimental Laboratory for Auxo-Endocrinological Research, 20145 Milan, Italy

**Keywords:** obesity, metabolic syndrome, sphingolipids, IDF diagnostic criteria

## Abstract

Metabolic syndrome is nosographically defined by using clinical diagnostic criteria such as those of the International Diabetes Federation (IDF) ones, including visceral adiposity, blood hypertension, insulin resistance and dyslipidemia. Due to the pathophysiological implications of the cardiometabolic risk of the obese subject, sphingolipids, measured in the plasma, might be used to biochemically support the diagnosis of metabolic syndrome. A total of 84 participants, including normal-weight (NW) and obese subjects without (OB-SIMET−) and with (OB-SIMET+) metabolic syndrome, were included in the study, and sphingolipidomics, including ceramides (Cer), dihydroceramides (DHCer), hexosyl-ceramides (HexCer), lactosyl-ceramides (LacCer), sphingomyelins (SM) and GM3 ganglosides families, and sphingosine-1-phosphate (S1P) and its congeners, was performed in plasma. Only total DHCers and S1P were significantly higher in OB-SIMET+ than NW subjects (*p* < 0.05), while total Cers decreased in both obese groups, though statistical significance was reached only in OB-SIMET− (vs. NW) subjects (*p* < 0.05). When considering the comparisons of the single sphingolipid species in the obese groups (OB-SIMET− or OB-SIMET+) vs. NW subjects, Cer 24:0 was significantly decreased (*p* < 0.05), while Cer 24:1, DHCer 16:0, 18:0, 18:1 and 24:1, and SM 18:0, 18:1 and 24:1 were significantly increased (*p* < 0.05). Furthermore, taking into account the same groups for comparison, HexCer 22:0 and 24:0, and GM3 22:0 and 24:0 were significantly decreased (*p* < 0.05), while HexCer 24:1 and S1P were significantly increased (*p* < 0.05). After having analyzed all data via a PLS-DA-based approach, the subsequent determination of the VIP scores evidenced the existence of a specific cluster of 15 sphingolipids endowed with a high discriminating performance (i.e., VIP score > 1.0) among the three groups, including DHCer 18:0, DHCer 24:1, Cer 18:0, HexCer 22:0, GM3 24:0, Cer C24:1, SM 18:1, SM 18:0, DHCer 18:1, HexCer 24:0, SM 24:1, S1P, SM 16:0, HexCer 24:1 and LacCer 22:0. After having run a series of multiple linear regressions, modeled by inserting each sphingolipid having a VIP score > 1.0 as a dependent variable, and waist circumference (WC), systolic/diastolic blood pressures (SBP/DBP), homeostasis model assessment-estimated insulin resistance (HOMA-IR), high-density lipoprotein (HDL), triglycerides (TG) (surrogates of IDF criteria) and C-reactive protein (CRP) (a marker of inflammation) as independent variables, WC was significantly associated with DHCer 18:0, DHCer 24:1, Cer 18:0, HexCer 22:0, Cer 24:1, SM 18:1, and LacCer 22:0 (*p* < 0.05); SBP with Cer 18:0, Cer 24:1, and SM 18:0 (*p* < 0.05); HOMA-IR with DHCer 18:0, DHCer 24:1, Cer 18:0, Cer 24:1, SM 18:1, and SM 18:0 (*p* < 0.05); HDL with HexCer 22:0, and HexCer 24:0 (*p* < 0.05); TG with DHCer 18:1, DHCer 24:1, SM 18:1, and SM 16:0 (*p* < 0.05); CRP with DHCer 18:1, and SP1 (*p* < 0.05). In conclusion, a cluster of 15 sphingolipid species is able to discriminate, with high performance, NW, OB-SIMET− and OB-SIMET+ groups. Although (surrogates of) the IDF diagnostic criteria seem to predict only partially, but congruently, the observed sphingolipid signature, sphingolipidomics might represent a promising “biochemical” support for the clinical diagnosis of metabolic syndrome.

## 1. Introduction

A large number of animal studies suggest that some specific ceramides (Cers) are associated with the onset and progression of cardiovascular diseases (CVDs) [1]. Many Cers-mediated pathophysiological mechanisms have been identified: promotion of atherogenesis by aggregation and subendothelial retention of low-density lipoproteins (LDL) within the vessel wall, induction of insulin resistance and hepatic steatosis/steatohepatitis, activation of low-grade chronic inflammation with an accumulation of visceral adipose tissue, and stimulation of oxidative stress with endothelial dysfunction and blood hypertension [2,3,4,5]. This supports the view that Cers and related congeners, which are present at very low concentrations in the plasma in respect to cholesterol or triglycerides (TGs), play a causative role in the metabolic dysfunction that precedes cardiovascular events occurring in the obese subject [6].

In different animal models, pharmacological inhibition and genetic inactivation of enzymes driving sphingolipids synthesis and degradation have been demonstrated to ameliorate atherosclerosis, insulin resistance, hepatic steatosis, blood hypertension, cardiomyopathy and type 2 diabetes mellitus (T2DM) [7,8,9,10,11,12,13,14,15,16,17,18].

In human studies, Cers (and other sphingolipids) have been shown to accumulate in atherosclerotic plaques [3]. This pathological finding would explain the strong correlation between circulating Cers levels and future cardiovascular events, such as myocardial infarction and stroke [19,20,21,22,23]. Numerous additional clinical studies have also reported associations of serum Cers and other sphingolipids with insulin resistance, a well-known risk factor for T2DM and various CVDs in clinical practice [24,25,26,27,28].

Overall, these findings seem to support the existence of a pathophysiological link between derangement of sphingolipid metabolism and cardiometabolic dysfunction. Moreover, they would provide the basis for the clinical use of sphingolipid levels in the plasma as a biochemical test, particularly in the diagnosis and monitoring of obese subjects with metabolic syndrome [29]. Nonetheless, so far, clinical studies investigating this topic are lacking or very few.

Clinicians are well aware of the limitations of the diagnostic criteria for metabolic syndrome, used in clinical practice and periodically updated by qualified scientific associations such as the International Diabetes Federation (IDF) [30]. In this regard, some authors have (provocatively?) proposed their abolition [31]. Thus, with the need for new and more predictive diagnostic criteria, the aims of the present study were the following: (1) to determine the plasma sphingolipidomic profile in a group of normal-weight (NW) and obese subjects without (OB-SIMET−) and with (OB-SIMET+) metabolic syndrome; (2) to investigate the performance of each sphingolipid in the discrimination of the three groups; and (3) to establish the weighted contributions of IDF diagnostic criteria for metabolic syndrome (i.e., visceral adiposity, insulin resistance, blood hypertension and dyslipidemia) in the prediction of some selected sphingolipids [30].

We hypothesize that sphingolipidomics might become a useful tool for a “biochemical” diagnosis of metabolic syndrome.

## 2. Results

Table 1 reports the demographic, clinical, and biochemical characteristics of the study population, subdivided into three groups (i.e., NW, OB-SIMET− and SIMET+) for a total of 84 subjects. In short, body mass index (BMI), waist to hip (circumferences) ratio (WHR), fat-free mass (FFM) (%), fat mass (FM) (kg and %), systolic blood pressure (SBP), diastolic blood pressure (DBP), heart rate (HR), resting energy expenditure (REE), insulin, homeostasis model assessment-estimated insulin resistance (HOMA-IR), high-density lipoprotein (HDL), triglycerides (TG), and C-reactive protein (CRP) were significantly different in OB-SIMET− and OB-SIMET+ subjects when compared to the NW group (*p* < 0.05), the differences of FFM (kg) and Hb1Ac being statistically significant only in the comparison NW vs. OB-SIMET+ group (*p* < 0.05). SBP was significantly higher in the OB-SIMET+ than in NW subjects (*p* < 0.05).

The following plasma sphingolipid families were measured: Cers, dihydroceramides (DHCers), hexosyl-ceramides (HexCers), lactosyl-ceramides (LacCers), sphingomyelins (SMs) and GM3 ganglosides (GM3s), along with sphingosine-1-phosphate (S1P) and its congeners (sphingosine (sph), dihydro-sphingosine (DHsph), dihydrosphingosine-1-phosphate (DHS1P)). Table 2 reports the concentrations and the statistically significant comparisons for all the sphingolipids among the three groups (*p* < 0.05). Cer 22:0, 24:0, SM 18:0, 18:1, HexCer 22:0, 24:0, 24:1, GM3 22:0 and 24:0 were significantly different in OB-SIMET− and OB-SIMET+ subjects when compared to the NW group (*p* < 0.05). In short, total Cers were lower in obese than in NW subjects, statistical significance being reached only in the OB-SIMET− group (*p* < 0.05). While Cer 22:0 and 24:0 followed this trend, Cer 24:1 increased in both obese groups, reaching statistical significance only in OB-SIMET+ subjects compared to NW (*p* < 0.05). Total DHCers and DHCer 16:0, 18:0, 18:1 and 24:1 all increased in both obese groups, statistical significance being reached in OB-SIMET+ subjects compared to NW; SM 18:0, 18:1 and 24:1, and S1P were significantly higher in OB-SIMET+ (but not OB-SIMET−) than in NW subjects (*p* < 0.05). HexCer 22:0 and 24:0, and GM3 22:0 and 24:0 were significantly lower in obese groups than in NW subjects, while, on the contrary, HexCer 24:1 was significantly increased (*p* < 0.05). See also the figures included in Supplementary Materials (Figures S1–S5).

**Figure 1:**
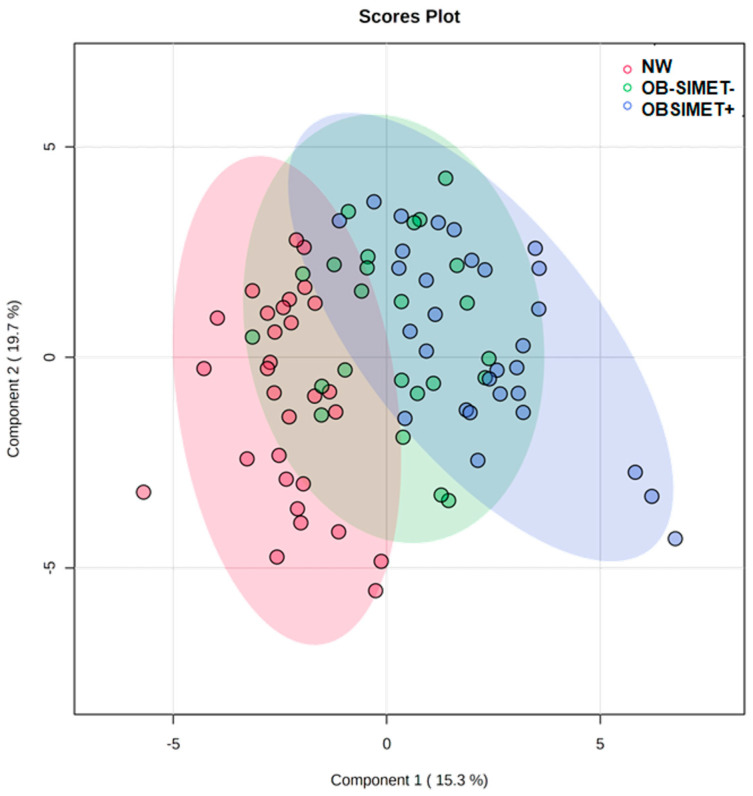
Comparison of the circulating lipidome in normal-weight (NW) and obese subjects without (OB-SIMET−) or with (OB-SIMET+) metabolic syndrome. Multivariate analysis—visualized as partial least squares discriminant analysis (PLS-DA)—of lipids in plasma for the three groups was used.

The PLS-DA-based approach, used to discriminate the sphingolipidomic profiles among the three groups (see Materials and Methods), showed a separation of 15.3% on the principal component (PC1), which represents a new dimension in which the initial variables (i.e., sphingolipids) are compressed, signifying the maximum separation that can be reached within these variables and clusters. Therefore, PLS-DA evidenced metabolic syndrome as a highly discriminating factor between lean and obese states (Figure 1).

**Figure 2:**
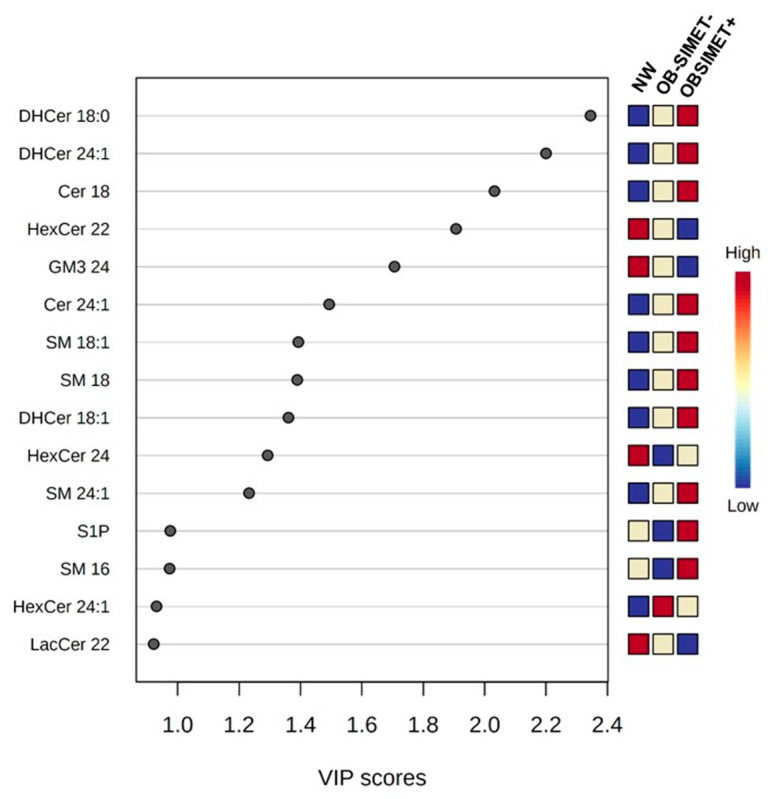
Graphic representation of the discriminating lipids, disclosed by high variance importance in projection (VIP) score, considering a cut-off ≥ 1.0.

The VIP scores (Figure 2), derived from PLS-DA, were used for ranking the discriminating features, taking a cut-off value > 1.0. From this analysis, a specific cluster of 15 sphingolipids endowed with high discriminating performance, hereinafter called discriminant sphingolipids, was identified as capable of marking univocally the differences among the three groups.

**Figure 3:**
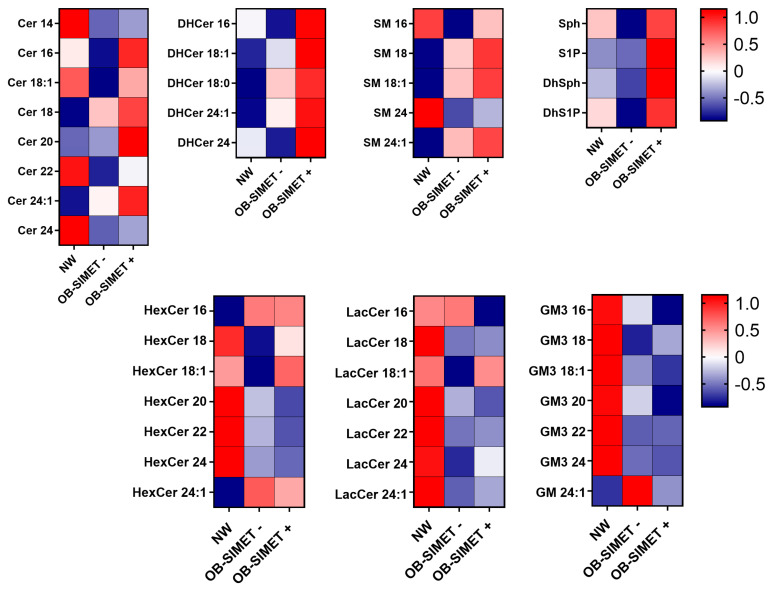
Visualization of sphingolipids as heatmaps. In particular, sphingolipid species were ordered according to their class—ceramides (Cer), dihydroceramides (DHCer), hexosyl-ceramides (HexCer), lactosyl-ceramides (LacCer), sphingomyelins (SM), GM3 ganglosides, and sphingoid bases—and visualized as heatmaps after transformation to z-values. The color-scale differentiates values as high (red), average (white), and low (blue).

In Figure 3, discriminant sphingolipids are visualized by heatmaps, evidencing clear-cut differences among NW, OB-SIMET− and OB-SIMET+ subjects. In some sphingolipid families, the different features showed a consistent pattern, i.e., a general decrease in plasma levels of HexCers, LacCers, and GM3 (with some exceptions), and a general increase in plasma levels of DHCers (with some exceptions), and sphingoid bases from lean to obese states. By contrast, the individual Cer species showed an opposite trend, some of them “*in crescendo*” and some of them “*in diminuendo*”.

Table 3 reports the results of the multiple linear regressions, modeled by inserting each sphingolipid having a VIP score > 1.0 as a dependent variable and the clinical parameters as independent variables. Among these, WC, SBP, HOMA-IR, HDL, TG, and CRP were significantly associated with several different sphingolipid species.

## 3. Discussion

Plasma levels of Cers and DHCers are increased in obesity, as reported by a large number of animal and human studies [32].

Apart from the increased plasma levels of Cer 18:0, in the present study plasma levels of different species of the DHCer family were higher in OB-SIMET− and/or OB-SIMET+ subjects when compared to the NW group (i.e., DHCer 16:0, 18:0, 18:1 and 24:1 and total DHCers).

For some time, increased availability of the substrates palmitate and serine was supposed to be the principal cause of the high plasma levels of Cers and DHCers in obesity. More recently, many other mechanisms, including low-grade chronic inflammation, oxidative stress, hormonal factors, and microbiome alterations, have been recognized to influence sphingolipid synthesis and degradation in obesity and related metabolic disorders [5]. Briefly, according to the most modern view, excessive consumption of saturated free fatty acids (FFAs), due to an unhealthy diet, stimulates toll-like receptor 4 (TLR4) signaling, leading to transcriptional activation of Cer biosynthetic genes, including Sptlc2 (serine palmitoyl transferase long chain base subunit 2) and specific CerS (ceramide synthase) isoforms [33,34,35]. Additionally, activation of intestinal hypoxia-inducible factor 2a (HIF-2a) during obesity has been demonstrated to contribute to hepatic steatosis by promoting Cers/DHCers accumulation, mainly due to the degradation of complex sphingolipids in the so-called salvage pathway [36], including a variety of HexCers, which, in the present study, were (not surprisingly) decreased (e.g., HexCers 22:0 and 24:0), suggesting a diversion towards Cers/DHCers conversion.

In the present study, plasma levels of S1P were higher in OB-SIMET+ than in NW and OB-SIMET− subjects, confirming the well-known link between S1P and metabolic syndrome [37]. As DHCers/Cers are hydrolyzed by ceramidases to sphingosine, which undergoes phosphorylation by a series of sphingosine kinases (SphKs) to form S1P, which may be considered a postulated protective mechanism to reduce lipo-toxicity in case of sphingolipid burden [32], a parallel increase in plasma DHCer/Cer and S1P is likely to occur in obesity and, particularly, in metabolic syndrome.

When data from our study population were collectively analyzed by a PLSDA-based approach, sphingolipidomics allowed discrimination between the three groups with high performance, especially between NW and OB-SIMET+ subjects. To corroborate this finding, the subsequent determination of the VIP score identified a specific cluster of sphingolipid species, including DHCer 18:0, DHCer 24:1, Cer 18:0, HexCer 22:0, GM3 24:0, Cer 24:1, SM 18:1, SM 18:0, DHCer 18:1, HexCer 24:0, SM 24:1, S1P, SM 16:0, HexCer 24:1, and LacCer 22:0.

The final aim of the present study being to define the weighted contribution of the IDF diagnostic criteria for metabolic syndrome [30], the prediction of each sphingolipid with a VIP score > 1.0 was calculated by inserting, in a model of linear regression, the clinical independent variables of our patients. An adjunctive parameter was considered, i.e., CRP, which, though it is not an IDF criterion, is a well-known marker of inflammation (not only) in obesity [38].

WC, which is defined as the “necessary” IDF criterion for the diagnosis of metabolic syndrome, and is a gross, but clinically practical surrogate of visceral obesity [30], in the present study was associated with a long list of sphingolipids, particularly: DHCer 18:0, DHCer 24:1, Cer 18:0, HexCer 22:0, Cer 24:1, SM 18:1 and LacCer 22:0, all with a VIP score > 1.0. A wealth of evidence has emerged demonstrating the molecular mechanisms underlying the link between visceral adiposity (i.e., WC) and synthesis/degradation of sphingolipids [5,32].

Adiponectin is an anti-diabetogenic and cardioprotective adipokine, which is reduced in the plasma of obese subjects, particularly those with high BMI and WC [39]. Interestingly, adiponectin receptors (particularly ADIPOR1 and ADIPOR2) show strong homology to intracellular ceramidases [40], suggesting that adiponectin is capable of preventing Cer accumulation when FFAs are needed for energy production, and explaining the inverse correlation between adiponectin and circulating and peripheral Cer in insulin-resistant individuals [28,41]. Therefore, the negative association between adiponectin and visceral adiposity (or WC) implies the positive association between sphingolipids and WC.

Although other reasons may be invoked to explain the association of WC with a series of sphingolipids having a high VIP score, such as the Cer-mediated inhibition of the hormone-sensitive lipase (HSL), expressed in adipose tissue [42], we believe that the low-grade chronic inflammation that typically characterizes visceral obesity [43] is the main pathophysiological linker of WC with sphingolipid metabolism [32]. Indeed, visceral adipose tissue releases a huge number of cytokines, including tumor necrosis factor α (TNF-α) or interleukin 6 (IL-6), which upregulate enzymes involved in the de novo Cer biosynthesis pathway (see also above) and activate sphingomyelinases [26,33,44,45,46].

The association of CRP with DHCer 18:1 and S1P, two sphingolipids having a VIP score > 1.0, is congruent with the view of an inflammation-mediated upregulation of genes coding enzymes involved in the biosynthesis of sphingolipids, and of an immunomodulatory role of some sphingolipids [32]. In this regard and focusing on S1P, despite some beneficial effects, SphKs and S1P exert deleterious functions, likely due to their known effects on immune cell trafficking and proinflammatory signaling [47]. Furthermore, similarly to the inflammation-driven activation of DHCer/Cer biosynthesis here-above described, saturated FFA overload upregulates SphK1 in the liver, where S1P in turn activates S1P receptor 1 (S1PR1) signaling in hepatocytes, leading to NF-kβ activation, elevated cytokine/chemokine production, and immune cell infiltration [48]. This cascade of events might explain the role of S1P in the pathogenesis of the non-alcoholic fatty liver disease (NAFLD), which is more prevalent in OB-SIMET+ than OB-SIMET− subjects [49].

In the present study, HOMA-IR, a surrogate of insulin resistance or T2DM, which represents an IDF diagnostic criterion for metabolic syndrome [30], was associated with many sphingolipids with the highest VIP scores (i.e., DHCer 18:0, DHCer 24:1, Cer 18:0, Cer 24:1, SM 18:1 and SM 18:0). In this regard, there is strong evidence linking derangement of sphingolipid metabolism, including plasma Cer/DHCer, to the development of insulin resistance [5,32]. This is supposed to be due to the blockade of insulin-stimulated AKT, a key serine/threonine kinase that regulates gluconeogenesis in the liver and glucose uptake in adipose and muscle tissue [11,50]. In particular, inhibition of AKT by Cer/DHCer is a consequence of the activation of two independent effectors, protein phosphatase 2A (PP2A) and protein kinase Cξ (PKCξ). Activation of PP2A causes dephosphorylation at T308 that inactivates AKT [51], while PKCξ, activated by Cer, phosphorylates T34 in a specific domain of AKT, preventing phosphatidylinositol-3,4,5-triphosphates (PIP3) from binding, and inhibiting AKT translocation and subsequent activation in response to insulin [51,52].

In the present study, TGs, another IDF diagnostic criterion of metabolic syndrome [30], was associated with DHCer 18:1, DHCer 24:1, SM 18:1 and SM 16:0, sphingolipids characterized by a high VIP score. Again, we can invoke the intervention of PKCξ to molecularly explain the link between sphingolipid metabolism and metabolic syndrome. Indeed, in the liver, PKCξ has been demonstrated to mediate the effect of Cer on the expression of sterol regulatory element binding transcription factor 1c (Srebp1c) [53], which plays a crucial role in the regulation of TGs biosynthesis. In addition, Srebp1c is implicated in the transcriptional regulation of the fatty acid translocase CD36, a multifunctional complex that facilitates the uptake of FFAs and enhances their esterification in TGs [41,54].

A recent study seems to provide another biochemical explanation for the association between TG and sphingolipid metabolisms, which progressively derange from morbid obesity to metabolic syndrome. In particular, hepatic Cer 16:0, specifically formed by CerS6, but not by CerS5, binds to mitochondrial fission factor (MFF), which, activated, promotes mitochondrial fission, causing mitochondrial dysfunction, an event that has been related to insulin resistance and obesity [55]. Some Cer species may also directly inhibit the Complex II and IV activity of mitochondrial electron transport [56]. This would block β-oxidation in the liver and adipose tissue, with subsequent increasing accumulation of TGs in lipid droplets, and with a possible spill-over in the plasma [15,57].

Among sphingolipids having a VIP score > 1.0, only HexCer 22:0 and 24:0, belonging to the group of glycosphingolipids, were associated with HDL, another IDF diagnostic criterion for metabolic syndrome 30].

The most abundant HexCers and LacCers are distributed on very-low-density lipoprotein (VLDL) (8–14%), LDL (46–60%) and HDL (28–44%) [58,59]. However, limited information is available about the origin of glycosphingolipids in plasma lipoproteins. Unlike SM and Cer, microsomal triglyceride transfer protein (MTP) is not implicated in the transfer of glycosphingolipids between lipoproteins (at least, in an in vitro model); furthermore, MTP deficiency in humans and animals does not affect plasma glycosphingolipids concentrations [60]. On the contrary, the main source of glycosphingolipids in the plasma has been attributed to HDL, although the mechanism(s) of efflux are as yet unidentified, with two possible (not demonstrated) transporters, ABCA12 or ABCC1 (where ABC refers to ATP-binding cassette) [61].

While the pathogenetic role of Cer and SM in atherogenesis has been widely recognized [62], to the best of our knowledge, the relationship between glycosphingolipids, particularly HexCer and LacCer, and atherogenesis (or, more extensively, CVD) has been investigated only in limited fashion. In the present study, plasma levels of HexCer 22:0 and 24:0 were decreased in OB-SIMET− and OB-SIMET+ subjects when compared to NW group and, in addition, were positively associated with HDL, implying a possible anti-atherogenic effect of these (or all?) glycosphingolipids. Apart from a structural role in pre-β-HDL discoid or (mature) HDL vesicles [61], the exact molecular function of HexCer 22:0 and 24:0 is missing, and further studies are mandatory to solve this issue, which might be crucial, due to the diagnostic and therapeutic implications.

In the present study, SBP (but not DBP) was negatively associated with Cer 18:0, Cer 24:1, and SM 18:0, i.e., plasma levels of Cer 24:1 and SM 18:0 higher in OB-SIMET+ (both molecules), and OB-SIMET− (only SM 18:0) than NW subjects. These findings might be difficult to interpret due to the general notion that alterations in sphingolipid metabolism are related to blood hypertension and other CVD outcomes [1]. Nevertheless, both animal and human studies have demonstrated a non-univocal link between plasma sphingolipids and endothelium-dependent vaso-regulation [63]. It is still controversial whether sphingolipids (and not only Cer) produce vasodilation or vasoconstriction effects. In particular, Cer 16:0 has been reported to produce vasoconstriction through the activation of a PCK-mediated pathway and, subsequently, an increase in Ca^2+^ entry into vascular smooth muscle cells [64]. On the contrary, S1P would produce vasodilatation, an effect mediated through activation of S1PR1 or S1PR3, which results in a stimulation of endothelial nitric oxide synthase (eNOS) and consequent release of endothelium-derived nitric oxide (NO), endowed with a potent vasorelaxant property [65]. Thus, the vascular tone is maintained by a balance between Cer and S1P, without ruling out other sphingolipids or different species of the same sphingolipid, which, so far, have not been fully investigated in terms of vaso-regulation [66]. These arguments may not only explain the results of the present study, but also open new areas of research for the treatment of blood hypertension [67].

Before closing, some limitations of the present study should be mentioned.

First of all, mass spectrometry technological advances over the last two decades have allowed us to have a more complex view of sphingolipid biochemistry as not one bulk substance but rather as a family of chemically and biologically distinct species [15]. Therefore, some of the conflicting (or difficult to be interpreted) results might be resolved by taking into account subcellular/tissue localization of sphingolipid and/or specific species, which might serve distinct functions. Furthermore, the induction of biosynthetic enzymes such as CerS isoforms under high-fat feeding or chronic inflammation may also contribute to the different biological effects (and results) observed [32].

Second, the gut microbiota has been demonstrated to contribute to the sphingolipidomic profile in the host’s plasma due to the ability of some bacterial species to synthesize and/or degrade sphingolipids. Therefore, we cannot rule out that the results of the present study may depend upon gut dysbiosis in OB-SIMET− or OB-SIMET+ subjects compared to the NW group [68].

Third, IDF diagnostic criteria for metabolic syndrome have been validated in a Caucasian population [30], from which subjects included in the present study were extracted. Our conclusions, particularly regarding sphingolipidomic signature, might be different when considering other ethnicities or exposomes.

Fourth, in the model of linear regression that was built in the present study, only a few independent variables were considered, i.e., (surrogates of) the IDF diagnostic criteria for metabolic syndrome, an obligatory choice due to our objectives. As widely discussed above, IDF diagnostic criteria only partially explain the high VIP score of some sphingolipids, along with the exclusion of others. The identification of the possible contributors (i.e., independent variables) is beyond the scope of the present study and deserves future investigation.

## 4. Materials and Methods

### 4.1. Subjects

Obese subjects (body mass index, BMI > 35 kg/m^2^), hospitalized at the Division of Metabolic Diseases, Istituto Auxologico Italiano, Piancavallo-Verbania, Italy, for a 3-week multidisciplinary integrated body weight reduction program (BWRP), were recruited for the current study. NW (healthy) subjects, age-matched, selected among friends and relatives of the medical and nursing staff, were recruited as the control group. Both obese and NW subjects were moderately active (60 min of physical activity, two times/week). All females were eumenorrheic; the study was carried out in the follicular phase of their menstrual cycle.

After having verified exclusion criteria, particularly the existence of any disease apart from essential obesity, or assumption of any drug, clinical, biochemical, and anthropometric data were collected from each participant, including evaluation of body composition by bioimpedance analysis (see below for details).

The study protocol was approved by the Ethical Committee (EC) of the Istituto Auxologico Italiano, IRCCS, Milan, Italy (EC code: 2021_02_23_11; research project: 01C126; acronym: SFINGOTRANSADIP); the protocol was explained to the subjects, who gave their written informed consent.

### 4.2. Anthropometric Measurements

A scale with a stadiometer was used to determine height and weight (Wunder Sa.Bi., WU150, Trezzo sull’Adda, Italy). Waist circumference (WC) was measured with a flexible tape in a standing position, halfway between the inferior margin of the ribs and the superior border of the crista, while hip circumference (HC) was measured at the largest parts around the buttocks. WC to HC ratio was consequently calculated (WHR). Body composition was measured by bioimpedance analysis (Human-IM Scan, DS-Medigroup, Milan, Italy) after 20 min of supine rest. BMI, fat mass (FM) and fat-free mass (FFM) were determined in all subjects.

### 4.3. Metabolic Variables

Blood samples (about 10 mL) were collected at around 8:00 a.m. after an overnight fast (about 12 h) at the beginning of the BWRP.

Total cholesterol (T-C), high-density lipoprotein cholesterol (HDL-C), low-density lipoprotein cholesterol (LDL-C), triglycerides (TG), glucose, insulin, and C-reactive protein (CRP) were measured.

Colorimetric enzymatic assays (Roche Diagnostics, Monza, Italy) were used to determine serum T-C, LDL-C, HDL-C, and TG levels. The sensitivities of the assays were 3.86 mg/dL (1 mg/dL = 0.03 mmol/L), 3.87 mg/dL (1 mg/dL = 0.03 mmol/L), 3.09 mg/dL (1 mg/dL = 0.03 mmol/L) and 8.85 mg/dL (1 mg/dL = 0.01 mmol/L), respectively.

Serum glucose level was measured by the glucose oxidase enzymatic method (Roche Diagnostics, Monza, Italy). The sensitivity of the method was 2 mg/dL (1 mg/dL = 0.06 mmol/L).

Serum insulin concentration was determined by a chemiluminescent immuno-metric assay, using a commercial kit (Elecsys Insulin, Roche Diagnostics, Monza, Italy). The sensitivity of the method was 0.2 µIU/mL (1 µU/mL = 7.18 pmol/L).

CRP was measured using an immunoturbidimetric assay (CRP RX, Roche Diagnostics GmbH, Mannheim, Germany). The sensitivity of the method was 0.03 mg/dL.

The intra- and inter-assay coefficients of variation (CVs) were the following: 1.1% and 1.6% for T-C, 1.2% and 2.5% for LDL-C, 1.8% and 2.2% for HDL-C, 1.1% and 2.0% for TG, 1.0% and 1.3% for glucose, and 1.5% and 4.9% for insulin.

For each patient, we also calculated the homeostatic model assessment of insulin resistance (HOMA-IR) according to the following formula: (insulin (μIU/mL) × glucose (mmol/L))/22.5 [69].

### 4.4. Blood Pressure

Blood pressure was measured on the right arm, using a sphygmomanometer with appropriate cuff size, with the subject in a seated position and a relaxed condition. The procedure was repeated three times at 10 min intervals; the means of the three values for systolic (SBP) and diastolic (DBP) blood pressures were recorded.

### 4.5. Definition of Metabolic Syndrome

According to the IDF criteria for diagnosis of metabolic syndrome in adults [30], obese patients were considered positive for the presence of metabolic syndrome if they had three or more of the following factors: (i) abdominal obesity; (ii) hypertriglyceridemia or specific treatment for this lipid abnormality; (iii) reduced HDL-C levels or specific treatment for this lipid abnormality; (iv) blood (systolic or diastolic) hypertension or treatment of previously diagnosed hypertension; (v) hyperglycemia or previously diagnosed T2DM.

### 4.6. Lipid Extraction and Sphingolipid Content Quantification

Sphingolipids extraction and targeted LC–MS/MS analysis were performed as previously described [70,71]. Sphingolipids were assayed in 25 µL of plasma, collected as described. Plasma was diluted to a final volume of 100 µL with water and, after the addition of 850 µL methanol/chloroform mixture (2:1 *v*/*v*), samples were incubated for 1 h at 38 °C. Then, to enhance their recovery, alkaline methanolysis was performed by incubation at 37 °C for 2 h with 75 µL of potassium hydroxide 1 M in methanol. After neutralization with 4 µL of pure acetic acid, samples were centrifuged (15 min at 13,400 RPM) and evaporated. The residues were dissolved in 100 µL of methanol, centrifuged for 10 min at 13,400 RPM, and withdrawn in a glass vial. Finally, samples were analyzed by LC Dionex 3000 UltiMate (ThermoFisher Scientific, Waltham, MA, USA) coupled to a tandem mass spectrometer AB Sciex 3200 QTRAP (AB Sciex, Framingham, MA, USA). The separation was achieved by reversed-phase chromatography either using BEH C8 1.7 μm, 100 mm × 2.1 mm (for ceramides, dihydroceramides, and sphingomyelins) or Cortecs C18 1.6 μm, 100 mm × 2.1 mm (Waters, Milford, MA, USA). (for sphingoid bases) by mixing eluent A (0.2% formic acid 2 mM ammonium formate water-solution) and eluent B (methanol 0.2% formic acid 1 mM ammonium formate). Quantitative analysis was performed interpolating each peak area of analyte/area IS with a calibration curve for each sphingolipid.

### 4.7. Statistics

Sigma Stat 4.0 (SysStat Software Inc., Palo Alto, CA, USA), Python 3.5 (Library, Scikit-learn), and GraphPad PRISM 7.0a (La Jolla, CA, USA) were used for analyses and plotting.

Parameters were expressed as median (interquartile range) and analyzed by Kruskal–Wallis’s one-way ANOVA test, followed by the post-hoc Dunn’s test for multiple comparisons (NW vs. OB-SIMET+ vs. OB-SIMET+). Categorical variables were compared through chi-square or Fisher tests.

Heat map representation was used to show, in a color-coded system, the concentrations of the sphingolipids within the three groups (NW, OB-SIMET−, and OB-SIMET+).

Partial least squares discriminant analysis (PLS-DA) was performed to increase the group separation (i.e., NW, OB-SIMET− and OB-SIMET+) and investigate the variables (i.e., sphingolipids) with a high Variance Importance in Projection score (VIP score  >  1.0).

Multiple linear regression analysis was performed to investigate the associations between each sphingolipid having a VIP score > 1.0 and some continuous clinical or biochemical variables (i.e., WC, SBP, DBP, HOMA-IR, TG, and HDL, which represent surrogates of IDF diagnostic criteria of metabolic syndrome [30], and CRP, which is a gross index of low-grade chronic inflammation in obesity [38]).

A *p*-value < 0.05 was considered statistically significant.

## 5. Conclusions

The present study carried out in a population of NW, OB-SIMET− and OB-SIMET+ subjects, has allowed identification of a (small) cluster of sphingolipid species able to discriminate, with a high performance, the three groups. The IDF diagnostic criteria for metabolic syndrome (i.e., WC, SBP/DBP, HOMA-IR, HDL, and TG) seem to predict only partially, but congruently, the observed sphingolipid signature, which, nevertheless, represents a promising “biochemical” support for the clinical diagnosis of metabolic syndrome. Finally, sphingolipidomic profiling of the OB-SIMET+ subject might be a molecular signature of pathophysiological mechanisms that are relevant in the development and/or worsening of metabolic syndrome, but that nosographically escape from the simplified clinical diagnostic criteria such as the IDF ones.

## Figures and Tables

**Table 1:** Demographic, clinical and biochemical characteristics of the study population, subdivided into three groups: normal-weight (NW) and obese subjects without (OB-SIMET−) or with (OB-SIMET+) metabolic syndrome.

Parameter	NW	OB-SIMET−	OB-SIMET+
N.	30	24	30
Sex (F/M)	19F-11M	18F-6M	19F-11M
Age (years)	29.15 [26.46; 33.14]	27.38 [21.35; 35.65]	30.43 [23.98; 41.18]
BMI (kg/m^2^)	22.85 [20.79; 24.70]	42.88 [40.75; 119.25] ^a^	43.44 [41.53; 46.54] ^a^
WHR	78 [76.25; 82.75]	110 [106; 82.75] ^a^	120 [113.25; 126.50] ^a^
FFM (kg)	53.06 [46.26; 59.02]	55.64 [50.84; 63.59]	61.86 [53.76; 66.03] ^a^
FFM %	79.45 [73.98; 82.30]	47.40 [44.88; 53.08] ^a^	48.75 [43.68; 52.73] ^a^
FM (kg)	13.13 [10.83; 17.95]	60.27 [52.80; 67.06] ^a^	61 [57.27; 68.08] ^a^
FM %	20.20 [17.53; 26.03]	52.60 [46.93; 55.13] ^a^	51.25 [47.40; 56.53] ^a^
SBP (mmHg)	120 [110; 120]	120 [120; 130] ^a^	130 [130; 140] ^a,b^
DBP (mmHg)	70 [70; 75]	80 [77.50; 80] ^a^	80 [80; 90] ^a^
HR (bm)	70 [69; 72]	80 [77.50; 90] ^a^	88 [84.25; 96.75] ^a^
REE (kcal/24 h)	1572.50 [1378.75; 1845.75]	1902.50 [1804; 2245.75] ^a^	2070.50 [1852.25; 2308.50] ^a^
Glucose (mg/dL)	87 [82.25; 94.25]	83 [80; 88.25]	86 [82.25; 94.75]
Insulin (mU/L)	6.65 [5.13; 8.80]	15.85 [11; 23.55] ^a^	25.05 [19.08; 30.25] ^a^
HOMA-IR	1.54 [1.07; 1.84]	3.23 [2.23; 4.68] ^a^	5.30 [4.32; 6.21] ^a^
T-C (mg/dL)	173 [158; 200.50]	160.50 [133.25; 188.50]	163 [148; 196]
HDL-C (mg/dL)	65 [56.25; 70.75]	45.50 [39.50; 50.25] ^a^	37.50 [32.50; 43.75] ^a^
LDL-C (mg/dL)	106.50 [86.25; 120.50]	101.50 [77.75; 122.25]	107.50 [96; 124.75]
TG (mg/dL)	63 [53; 85.75]	96 [85.75; 123.25] ^a^	125.50 [103.50; 159.25] ^a^
HbA1c (mmol/L)	5.10 [5; 5.30]	5.10 [5; 5.40]	5.40 [5.10; 5.60] ^a^
CRP (mg/dL)	0.10 [0; 0.20]	0.50 [0.28; 1.03] ^a^	0.55 [0.40; 1.08] ^a^

Note: Data, expressed as median and interquartile range (25th and 75th), were analyzed by Kruskal–Wallis’s one-way ANOVA test, followed by the post-hoc Dunn’s test for multiple comparisons. ^a^: <0.05 vs. NW group; ^b^: <0.05 vs. OB-SIMET−. For abbreviations, see the list included in the text.

**Table 2:** Plasma sphingolipidomics in the study population, subdivided into three groups: normal-weight (NW) and obese subjects without (OB-SIMET−) or with (OB-SIMET+) metabolic syndrome.

Sphingolipid	NW			OB-SIMET−			OB-SIMET+		
(µmol/L)	Median	25th	75th	Median	25th	75th	Median	25th	75th
Cer 14:0	0.0168	0.0140	0.0207	0.0124	0.0102	0.0174	0.0138	0.0112	0.0159
Cer 16:0	0.4463	0.3834	0.5280	0.4359	0.4025	0.4727	0.4533	0.3815	0.5484
Cer 18:1	0.0149	0.0132	0.0171	0.0143	0.0123	0.0164	0.0156	0.0127	0.0176
Cer 18:0	0.0663	0.0557	0.0790	0.1121	0.0794	0.1377	0.1242	0.1053	0.1714
Cer 20:0	0.0810	0.0640	0.0961	0.0861	0.0679	0.0968	0.0859	0.0718	0.1213
Cer 22:0	0.6195	0.4530	0.7052	0.4103 ^a^	0.3459	0.5539	0.4815	0.3658	0.6725
Cer 24:1	0.7663	0.5601	0.9362	0.9581	0.8408	1.1890	1.1021 ^a^	0.8999	1.3634
Cer 24:0	3.7086	2.9225	4.0040	2.1955 ^a^	1.7563	3.1066	2.3265 ^a^	1.9734	3.0387
DHCER 16:0	0.0260	0.0200	0.0356	0.0240	0.0204	0.0282	0.0335 ^b^	0.0250	0.0402
DHCer 18:1	0.0046	0.0031	0.0075	0.0061	0.0046	0.0100	0.0084 ^a^	0.0069	0.0128
DHCer 18:0	0.0060	0.0037	0.0078	0.0170 ^a^	0.0108	0.0205	0.0230 ^a^	0.0177	0.0323
DHCER 24:1	0.0746	0.0542	0.1108	0.1415 ^a^	0.0931	0.1991	0.2081 ^a,b^	0.1541	0.2759
DHCer 24:0	0.1817	0.1339	0.2618	0.1630	0.1248	0.2486	0.2486	0.1697	0.3262
SM 16:0	120.4211	113.9908	131.7082	117.3254	101.4098	127.1680	121.3630	110.4303	133.8067
SM 18:0	33.2488	24.8709	38.5798	41.0255 ^a^	31.5397	47.9056	47.6799 ^a^	39.0624	54.0556
SM 18:1	21.7878	18.5967	24.0871	28.0090 ^a^	25.9735	31.1825	31.2947 ^a^	28.3160	34.2958
SM 24:0	17.1339	11.9928	28.2404	13.4373	10.2122	21.6739	14.3467	10.0835	21.3914
SM 24:1	40.4368	29.6155	49.6716	50.2870	37.7062	59.8301	48.4161 ^a^	42.1587	59.6591
Total Cer	5.8230	4.3690	6.3426	4.3506 ^a^	3.7056	5.5548	4.5706	3.8788	6.0811
Total DHCer	0.3034	0.2126	0.4238	0.3677	0.2625	0.4987	0.5083 ^a,b^	0.4007	0.7113
Total SM	237.6740	204.1032	265.0412	246.9296	221.2554	279.2727	270.6917	234.2189	304.6080
HexCer 16:0	1.4962	1.2149	1.8136	1.3930	1.2467	1.7919	1.4189	1.1997	1.8053
HexCer 18:0	0.2198	0.1820	0.2800	0.2269	0.2033	0.2564	0.2253	0.1839	0.2572
HexCer 18:1	0.0288	0.0167	0.0451	0.0209	0.0163	0.0276	0.0347	0.0182	0.0462
HexCer 20:0	0.2955	0.2324	0.3929	0.2625	0.2014	0.3078	0.2503	0.1916	0.2917
HexCer 22:0	4.1124	3.7338	5.0359	2.8256 ^a^	2.3509	3.4540	2.7980 ^a^	2.0631	3.3255
HexCer 24:0	4.6624	3.8590	5.7986	3.1278 ^a^	2.3262	4.0775	3.0659 ^a^	2.3020	4.7563
HexCer 24:1	3.8078	3.3694	4.7813	5.2074 ^a^	4.4501	6.6098	5.0210 ^a^	4.0688	6.5814
LacCer 16:0	8.1071	6.6496	10.3397	7.8664	6.4482	10.4543	7.0130	6.0758	8.9284
LacCer 18:0	0.1483	0.1045	0.2188	0.1370	0.0964	0.1783	0.1199	0.0883	0.2107
LacCer 18:1	0.0454	0.0361	0.0709	0.0421	0.0320	0.0632	0.0478	0.0328	0.0616
LacCer 20:0	0.0519	0.0267	0.1098	0.0430	0.0243	0.0774	0.0300	0.0194	0.0648
LacCer 22:0	0.1005	0.0648	0.4088	0.1216	0.0385	0.2006	0.0519	0.0276	0.1925
LacCer 24:0	0.0154	0.0065	0.1763	0.0397	0.0049	0.1195	0.0089	0.0032	0.0940
LacCer 24:1	0.2026	0.1410	1.3683	0.3930	0.1163	1.1021	0.1191	0.0701	0.8776
GM3 16:0	2.1501	1.6216	3.0089	1.8378	1.6095	2.6726	1.7537	1.3393	2.2612
GM3 18:0	0.4925	0.3363	0.7387	0.3964	0.2763	0.6066	0.3483	0.2703	0.4925
GM3 18:1	0.0240	0.0240	0.0480	0.0240	0.0240	0.0300	0.0240	0.0000	0.0480
GM3 20:0	0.1201	0.0541	0.1922	0.1081	0.0661	0.1682	0.0841	0.0480	0.1201
GM3 22:0	1.2252	0.8108	1.6996	0.7447 ^a^	0.4985	0.9429	0.6246 ^a^	0.4444	0.7928
GM3 24:0	0.2162	0.1742	0.3123	0.1201 ^a^	0.0480	0.1501	0.0721 ^a^	0.0240	0.1201
GM3 24:1	1.0991	0.8108	1.3453	1.2132	0.7087	1.7837	1.0450	0.8288	1.4579
Total HexCer	14.6067	12.9005	16.8163	13.5736	11.3378	15.6432	12.7833	10.3120	16.5317
Total LacCer	8.8129	7.0560	13.4106	9.3226	6.7950	12.6217	7.3023	6.2825	10.8854
Total GM3	5.1409	3.8797	6.4382	4.3722	3.0855	5.8496	3.9037	3.2011	4.7520
Sph	0.1182	0.0982	0.1528	0.1094	0.0977	0.1233	0.1207	0.1059	0.1483
S1P	1.6429	1.3007	1.9686	1.5321	1.3267	1.7749	1.9854 ^a,b^	1.7742	2.1176
DHSph	0.0155	0.0132	0.0204	0.0177	0.0131	0.0207	0.0187	0.0133	0.0242
DHS1P	0.3589	0.2552	0.4163	0.2694	0.2282	0.3673	0.3509	0.2894	0.4011

Note: Data, expressed as median and interquartile range (25th and 75th), were analyzed by Kruskal–Wallis’s one-way ANOVA test, followed by the post-hoc Dunn’s test for multiple comparisons. ^a^: <0.05 vs. NW group; ^b^: <0.05 vs. OB-SIMET−. The background color highlights the statistical significance. For abbreviations, see the list included in the text.

**Table 3:** Multiple linear regression of each sphingolipid (dependent variable) with surrogates of IDF diagnostic criteria for metabolic syndrome (independent variables), i.e., WC, SBP/DBP, HOMA-IR, HDL, and TG, including CRP.

	Coefficient (×10^−3^)	Std. Error (×10^−3^)	t	*p*	VIF
**DHCer 18:0**					
Constant	−4.7	11.4	−0.409	0.684	
WC (cm)	0.3	0.1	3.747	**<0.001**	3.476
SBP (mmHg)	−0.1	0.1	−0.779	0.439	2.478
DBP (mmHg)	−0.2	0.1	−1.281	0.204	2.291
HOMA-IR	2.0	0.4	4.739	**<0.001**	1.864
HDL (mg/dL)	0.1	0.1	0.777	0.44	2.583
TG (mg/dL)	0.0	0.0	2.122	0.037	1.567
CRP (mg/dL)	2.0	1.6	1.294	0.2	1.38
**DHCer 24:1**					
Constant	19.2	97.4	0.198	0.844	
WC (cm)	1.5	0.6	2.408	0.018	3.476
SBP (mmHg)	−0.6	0.8	−0.752	0.454	2.478
DBP (mmHg)	−1.0	1.1	−0.943	0.349	2.291
HOMA-IR	15.2	3.6	4.183	**<0.001**	1.864
HDL (mg/dL)	0.4	0.7	0.65	0.518	2.583
TG (mg/dL)	0.6	0.2	3.504	**<0.001**	1.567
CRP (mg/dL)	12.8	13.4	0.953	0.344	1.38
**Cer 18:0**					
Constant	90.1	66.4	1.356	0.179	
WC (cm)	1.0	0.4	2.459	**0.016**	3.476
SBP (mmHg)	−1.3	0.6	−2.25	**0.027**	2.478
DBP (mmHg)	0.6	0.8	0.801	0.426	2.291
HOMA-IR	6.4	2.5	2.571	**0.012**	1.864
HDL (mg/dL)	0.0	0.5	−0.0872	0.931	2.583
TG (mg/dL)	0.0	0.1	−0.0714	0.943	1.567
CRP (mg/dL)	15.4	9.1	1.683	0.096	1.38
**HexCer 22:0**					
Constant	4314.0	1757.0	2.455	**0.016**	
WC (cm)	−22.3	10.9	−2.057	**0.043**	3.476
SBP (mmHg)	−21.4	15.3	−1.395	0.167	2.478
DBP (mmHg)	30.1	19.8	1.517	0.133	2.291
HOMA-IR	65.3	65.7	0.994	0.323	1.864
HDL (mg/dL)	30.7	12.1	2.536	**0.013**	2.583
TG (mg/dL)	0.6	2.9	0.195	0.846	1.567
CRP (mg/dL)	−35.8	241.0	−0.148	0.883	1.38
**GM3 24:0**					
Constant	602.0	237.0	2.539	0.013	
WC (cm)	−2.0	1.5	−1.335	0.186	3.476
SBP (mmHg)	−3.4	2.1	−1.665	0.1	2.478
DBP (mmHg)	2.4	2.7	0.882	0.381	2.291
HOMA-IR	1.8	8.9	0.203	0.84	1.864
HDL (mg/dL)	0.8	1.6	0.466	0.643	2.583
TG (mg/dL)	−0.2	0.4	−0.485	0.629	1.567
CRP (mg/dL)	−19.7	32.6	−0.604	0.548	1.38
**Cer 24:1**					
Constant	562.0	460.0	1.222	0.226	
WC (cm)	7.9	2.8	2.765	**0.007**	3.476
SBP (mmHg)	−9.6	4.0	−2.384	**0.02**	2.478
DBP (mmHg)	4.0	5.2	0.777	0.439	2.291
HOMA-IR	37.8	17.2	2.201	**0.031**	1.864
HDL (mg/dL)	4.0	3.2	1.274	0.207	2.583
TG (mg/dL)	0.8	0.8	1.077	0.285	1.567
CRP (mg/dL)	65.4	63.2	1.034	0.304	1.38
**SM 18:1**					
Constant	5765.0	7455.0	0.773	0.442	
WC (cm)	167.0	46.1	3.633	**<0.001**	3.476
SBP (mmHg)	−96.1	65.0	−1.478	0.143	2.478
DBP (mmHg)	90.7	84.2	1.078	0.285	2.291
HOMA-IR	801.0	279.0	2.874	**0.005**	1.864
HDL (mg/dL)	71.4	51.3	1.392	0.168	2.583
TG (mg/dL)	27.1	12.3	2.208	**0.03**	1.567
CRP (mg/dL)	−1023.0	1024.0	−0.999	0.321	1.38
**SM 18:0**					
Constant	38,547.0	14,936.0	2.581	**0.012**	
WC (cm)	171.0	92.3	1.852	0.068	3.476
SBP (mmHg)	−333.0	130.0	−2.557	**0.013**	2.478
DBP (mmHg)	256.0	169.0	1.52	0.133	2.291
HOMA-IR	1359.0	558.0	2.435	**0.017**	1.864
HDL (mg/dL)	−14.6	103.0	−0.142	0.887	2.583
TG (mg/dL)	−0.1	24.6	−0.00211	0.998	1.567
CRP (mg/dL)	2107.0	2052.0	1.027	0.308	1.38
**DHCer 18:1**					
Constant	−5.6	7.9	−0.711	0.48	
WC (cm)	0.0	0.0	0.996	0.322	3.476
SBP (mmHg)	0.0	0.1	0.183	0.855	2.478
DBP (mmHg)	0.0	0.1	0.092	0.927	2.291
HOMA-IR	0.5	0.3	1.623	0.109	1.864
HDL (mg/dL)	0.1	0.1	1.058	0.293	2.583
TG (mg/dL)	0.0	0.0	0.195	0.846	1.567
CRP (mg/dL)	2.3	1.1	2.123	0.037	1.38
**HexCer 24:0**					
Constant	3487.0	2627.0	1.327	0.188	
WC (cm)	−18.2	16.2	−1.12	0.266	3.476
SBP (mmHg)	−31.1	22.9	−1.357	0.179	2.478
DBP (mmHg)	41.8	29.7	1.41	0.162	2.291
HOMA-IR	80.8	98.2	0.823	0.413	1.864
HDL (mg/dL)	45.5	18.1	2.519	**0.014**	2.583
TG (mg/dL)	4.6	4.3	1.051	0.296	1.567
CRP (mg/dL)	79.0	361.0	0.219	0.827	1.38
**SM 24:1**					
Constant	55,470.0	20,366.0	2.724	0.008	
WC (cm)	81.7	126.0	0.649	0.518	3.476
SBP (mmHg)	−329.0	178.0	−1.853	0.068	2.478
DBP (mmHg)	255.0	230.0	1.107	0.272	2.291
HOMA-IR	1387.0	761.0	1.822	0.072	1.864
HDL (mg/dL)	−67.3	140.0	−0.48	0.632	2.583
TG (mg/dL)	3.9	33.5	0.116	0.908	1.567
CRP (mg/dL)	3251.0	2798.0	1.162	0.249	1.38
**S1P**					
Constant	282.0	716.0	0.394	0.695	
WC (cm)	−3.0	4.4	−0.676	0.501	3.476
SBP (mmHg)	8.3	6.2	1.334	0.186	2.478
DBP (mmHg)	2.7	8.1	0.331	0.742	2.291
HOMA-IR	39.1	26.8	1.46	0.148	1.864
HDL (mg/dL)	3.4	4.9	0.69	0.493	2.583
TG (mg/dL)	0.9	1.2	0.772	0.443	1.567
CRP (mg/dL)	211.0	98.4	2.145	0.035	1.38
**SM 16:0**					
Constant	97,236.0	26,232.0	3.707	<0.001	
WC (cm)	58.5	162.0	0.361	0.719	3.476
SBP (mmHg)	−336.0	229.0	−1.471	0.145	2.478
DBP (mmHg)	429.0	296.0	1.447	0.152	2.291
HOMA-IR	151.0	980.0	0.154	0.878	1.864
HDL (mg/dL)	330.0	181.0	1.826	0.072	2.583
TG (mg/dL)	105.0	43.2	2.434	**0.017**	1.567
CRP (mg/dL)	−3135.0	3604.0	−0.87	0.387	1.38
**C24:1-HexCer**					
Constant	2457.0	2332.0	1.053	0.296	
WC (cm)	19.3	14.4	1.339	0.185	3.476
SBP (mmHg)	−35.0	20.3	−1.721	0.089	2.478
DBP (mmHg)	44.0	26.3	1.669	0.099	2.291
HOMA-IR	127.0	87.2	1.463	0.148	1.864
HDL (mg/dL)	16.1	16.0	1.001	0.32	2.583
TG (mg/dL)	−0.2	3.8	−0.0417	0.967	1.567
CRP (mg/dL)	311.0	320.0	0.97	0.335	1.38
**LacCer 22:0**					
Constant	522.0	334.0	1.56	0.123	
WC (cm)	−5.5	2.1	−2.653	**0.01**	3.476
SBP (mmHg)	−1.2	2.9	−0.415	0.679	2.478
DBP (mmHg)	5.2	3.8	1.364	0.176	2.291
HOMA-IR	12.2	12.5	0.976	0.332	1.864
HDL (mg/dL)	−0.9	2.3	−0.395	0.694	2.583
TG (mg/dL)	−0.4	0.6	−0.67	0.505	1.567
CRP (mg/dL)	46.7	45.9	1.017	0.312	1.38

Note: the bold highlights the statistical significance; for abbreviations, see the list included in the text.

## Data Availability

The datasets used and/or analyzed in the present study are available from the corresponding author upon a reasonable request. Raw data have been uploaded to https://doi.org/10.5281/zenodo.7833681 (accessed on 14 April 2023).

## Supplementary Materials

The following supporting information can be downloaded at: https://www.mdpi.com/article/10.3390/ijms24087451/s1.

## Abbreviations

ABC, ATP-binding cassette; ADIPOR1, adiponectin receptor 1; ADIPOR2, adiponectin receptor 2; ATP, adenosine triphosphate; BMI, body mass index; BWRP, body weight reduction program; Cer, ceramide; CerS, ceramide synthase; CRP, C-reactive protein; CVD, cardiovascular disease; DBP, diastolic blood pressure; DHCer, dihydroceramide; DHS1P, dihydrosphingosine-1-phosphate; DHsph, sphingosine (sph],dihydro-sphingosine; EC, Ethical Committee; eNOS, endothelial nitric oxide synthase; FFA, free fat acids; FFM, fat-free mass; FM, fat mass; GM3, GM3 gangloside; HbA1c, glycated hemoglobin; HC, hip circumference; HDL, high density lipoprotein; HexCer, hexosyl-ceramide; HIF-2a, hypoxia-inducible factor 2a; HOMA-IR, homeostasis model assessment of insulin resistance; HR, heart rate; HSL, hormone-sensitive lipase; IDF, International Diabetes Federation; IL-6, interleukine 6; LacCer, lactosyl-ceramide; LC–MS/MS, liquid chromatography–tandem mass spectrometry; LDL, low density lipoprotein; MFF, mitochondrial fission factor; MTP, microsomal triglyceride transfer protein; NAFLD, non-alcoholic fatty liver disease; NF-kβ, nuclear factor kβ; NO, nitric oxide; NW, normal-weight; OB-SIMET−, obese without metabolic syndrome; OB-SIMET+, obese with metabolic syndrome; PIP3, phosphatidylinositol-3,4,5-triphosphates; PKCξ, protein kinase Cξ; PLS-DA, partial least-squares-discriminant analysis; PP2A, protein phosphatase 2A; REE, resting energy expenditure; S1P, sphingosine-1-phosphate; S1PR1, S1P receptor 1; S1PR3, S1P receptor 3; SBP, systolic blood pressure; SM, sphingomyelin; Sph, sphingosine; SphK, sphingosine kinase; Sptlc2, serine palmitoyl transferase long chain base subunit 2; Srebp1c, sterol regulatory element binding transcription factor 1c; T, threonine; T2DM, type 2 diabetes mellitus; T-C, total cholesterol; TG, triglycerides; TLR4, toll-like receptor 4; TNF-α, tumor necrosis factor α; VIP, variance importance in projection; VLDL, very-low-density lipoprotein; WC, waist circumference; WHR, waist to hip ratio.
