# Supplementary material for: Identification of a Specific Plasma Sphingolipid Profile in a Group of Normal-Weight and Obese Subjects: A Novel Approach for a “Biochemical” Diagnosis of Metabolic Syndrome?
Source: Int J Mol Sci. 2023 Apr 18;24(8):7451. doi: 10.3390/ijms24087451 (PMC10138812; doi:10.3390/ijms24087451)
Supplement: Supplementary file 1 [file ijms-24-07451-s001.zip › LEGENDE to Figures S1-S2-S3-S4-S5.pdf]

Supplementary Figures S1-S2. Boxplots represent, for each lipid class, the concentrations of all the species (i.e., sum) measured in the plasma of NW (n=30), OB-SIMET- (N=30) and OB-SIMET+ (n=24) subjects. Lipids of a same class were summed and then mediated within all subjects of the same class. The boxes represent data obtained in the range 25th–75th percentiles; the line across the boxes indicates the median value; the lines above and below the boxes indicate extreme values (10th or 90th percentile). Statistical significance was evaluated by Kruskal–Wallis's one-way ANOVA test, followed by the *post-hoc* Dunn's test for multiple comparisons (NW vs. OB-SIMET+ vs. OB-SIMET+). a: <0.05 vs. NW group; b: <0.05 vs. OB-SIMET-. For abbreviations see the article.

Supplementary Figures S3-S4-S5. Boxplots represent the median (25th-75th percentiles) of the single lipid species reported in Table 3 that showed a significant variation by Kruskal–Wallis's one-way ANOVA test, followed by the *post-hoc* Dunn's test for multiple comparisons (NW vs. OB-SIMET+ vs. OB-SIMET+). a: <0.05 vs. NW group; b: <0.05 vs. OB-SIMET-. For the abbreviations see the article.
