# Supplementary figures and images for: Identification of a Specific Plasma Sphingolipid Profile in a Group of Normal-Weight and Obese Subjects: A Novel Approach for a “Biochemical” Diagnosis of Metabolic Syndrome?
Source: Int J Mol Sci. 2023 Apr 18;24(8):7451. doi: 10.3390/ijms24087451 (PMC10138812; doi:10.3390/ijms24087451)

## Slide 1
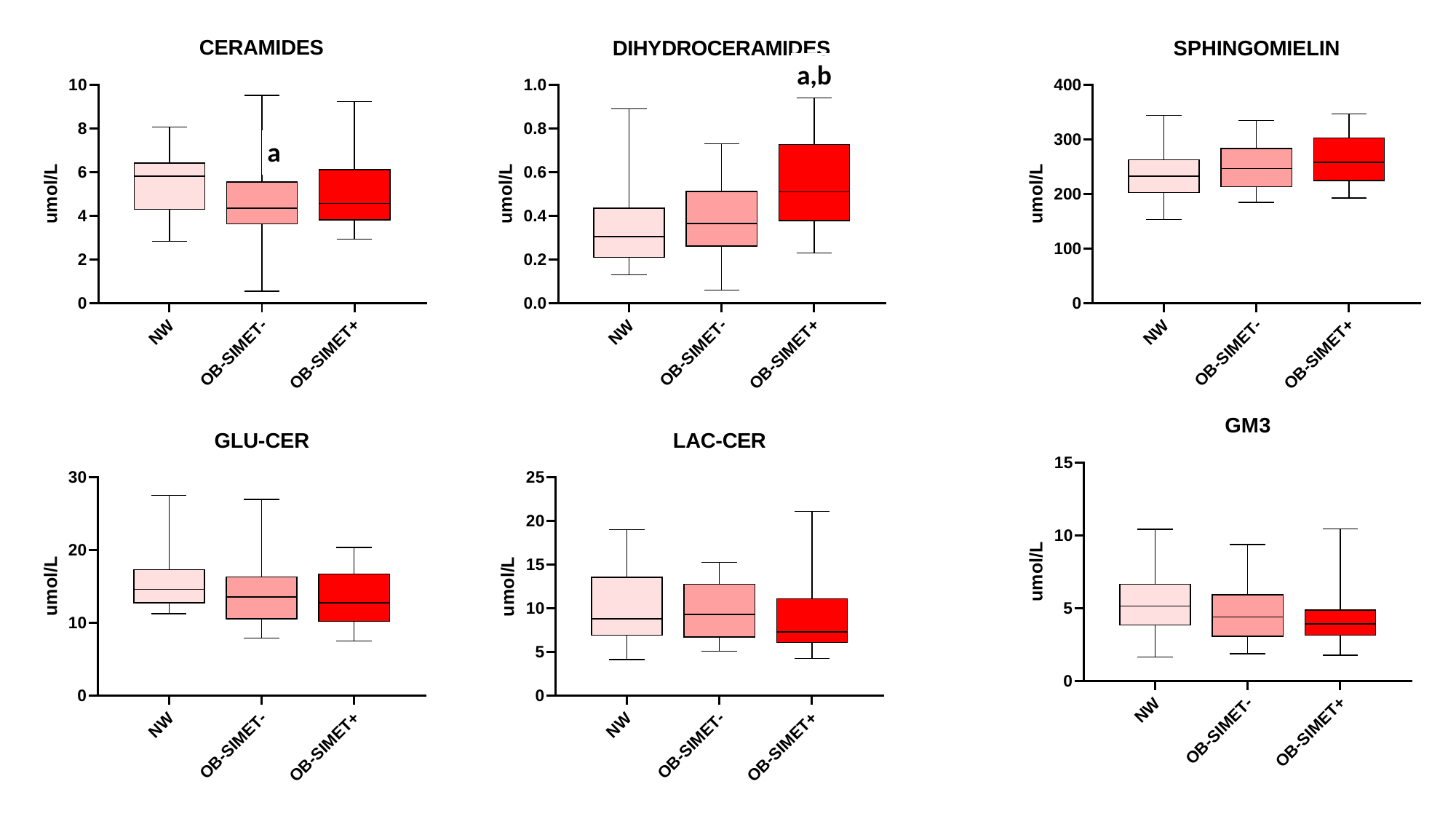

## Slide 2
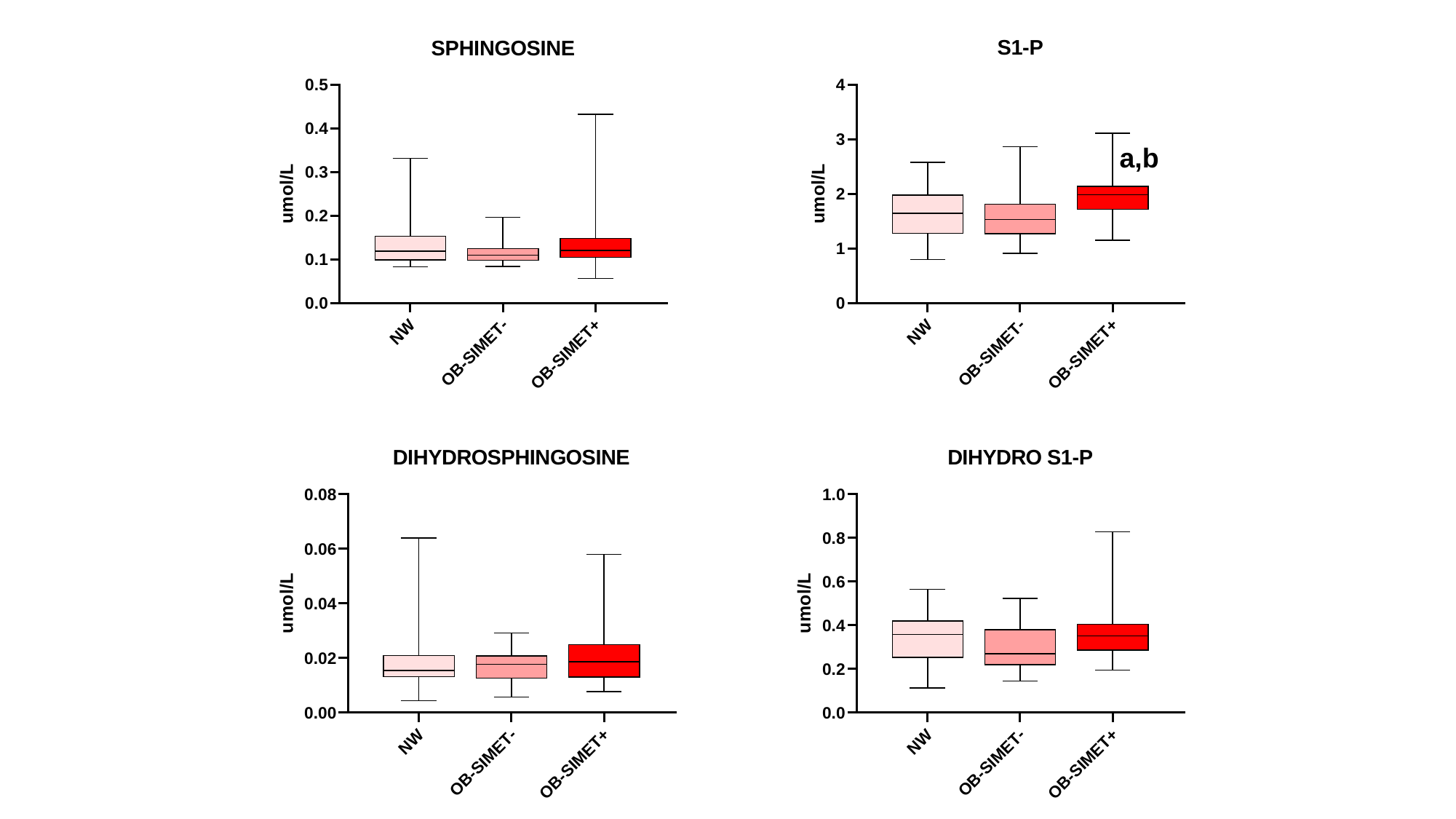

Supplement: Supplementary file 1 [file ijms-24-07451-s001.zip › Figures S1-S2.pptx]

## Slide 1
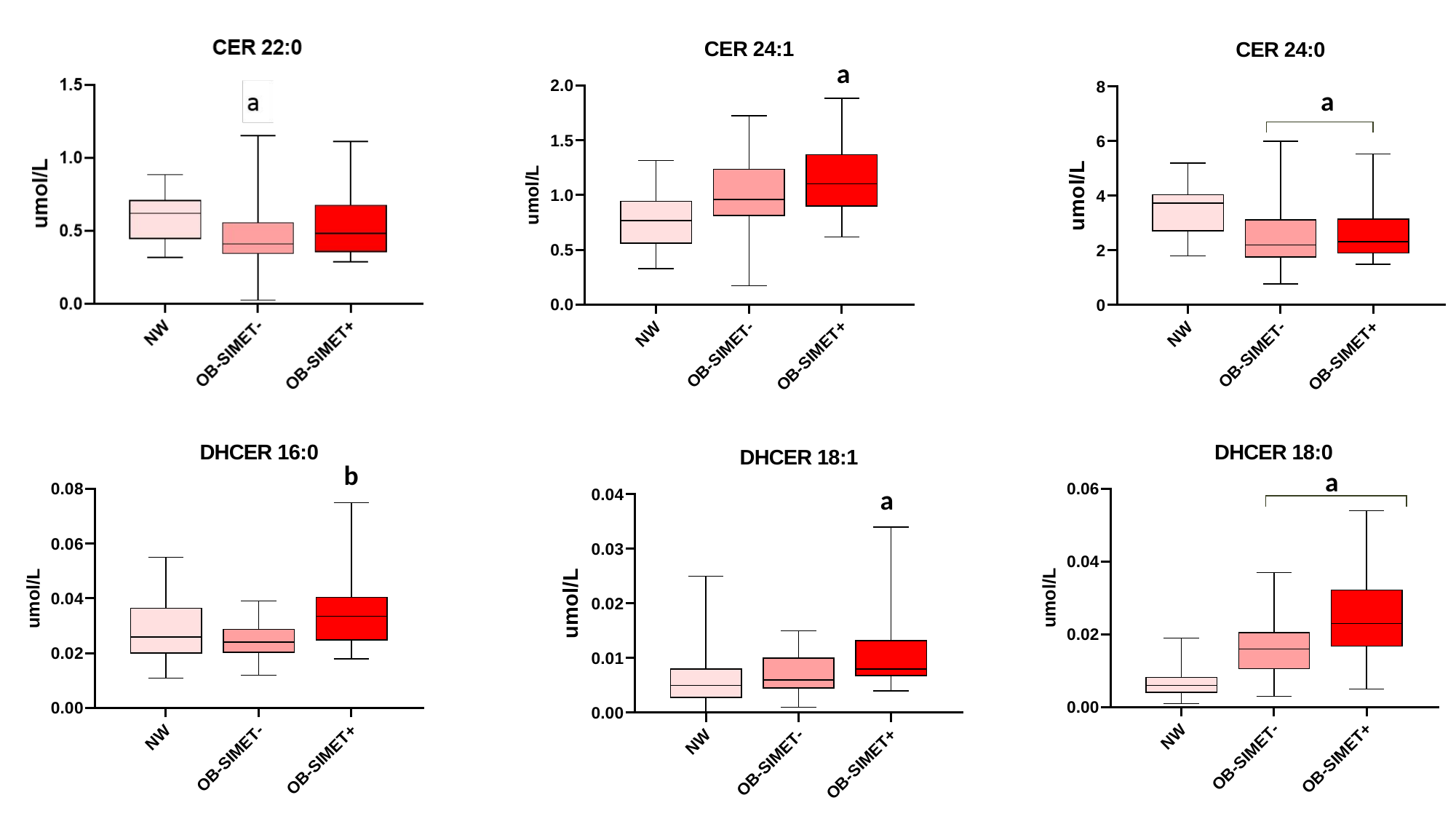

## Slide 2
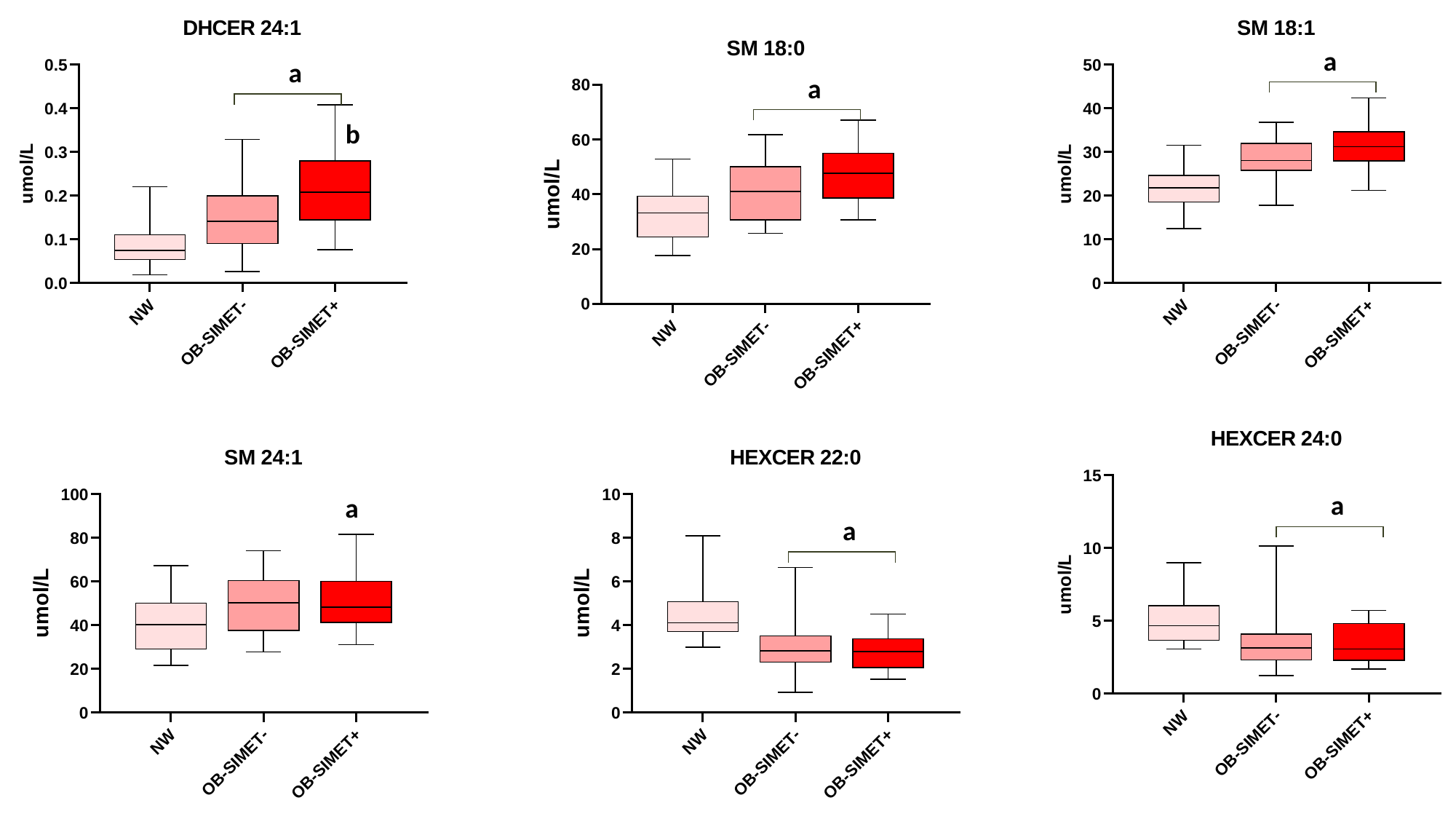

## Slide 3
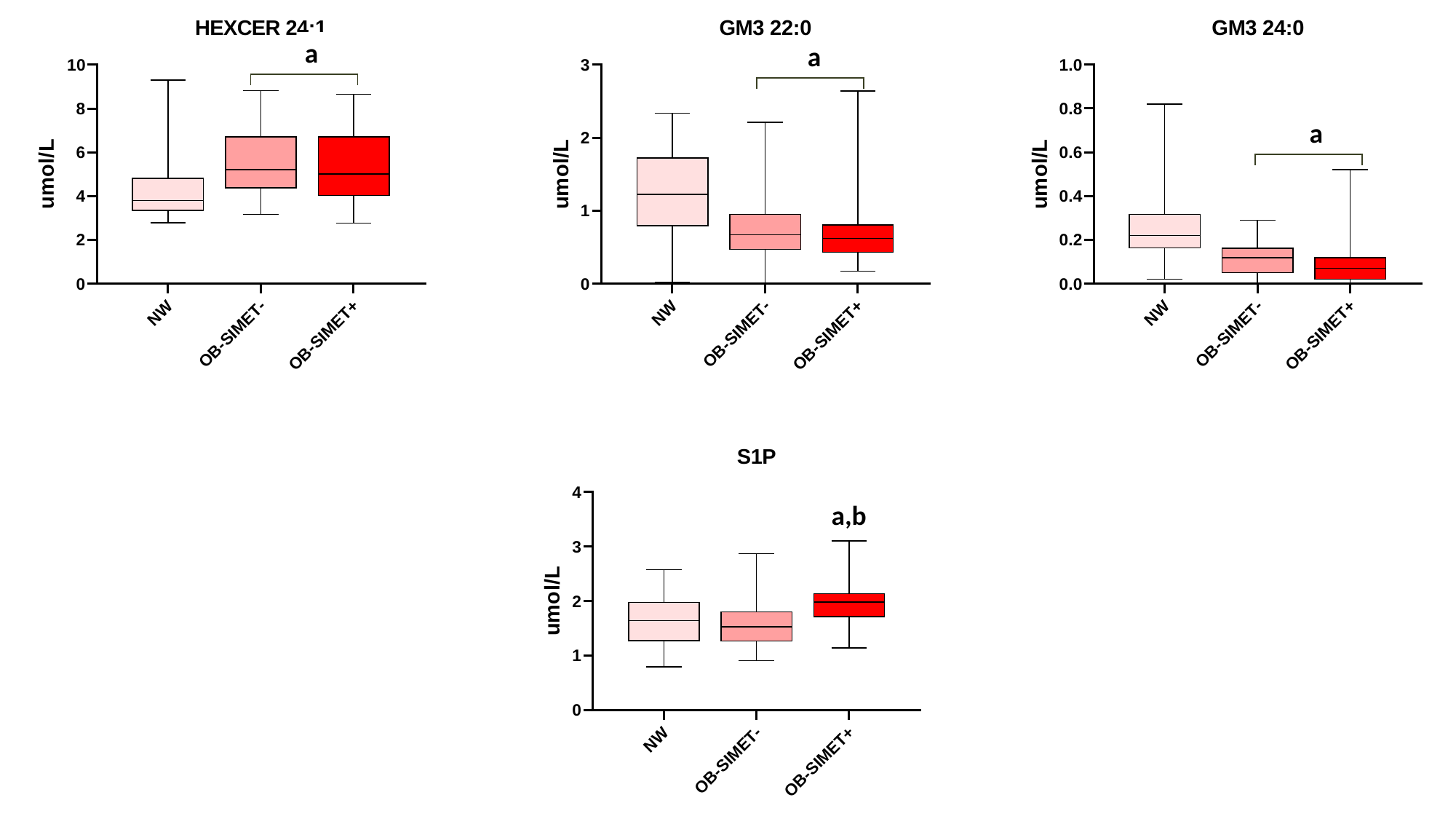

Supplement: Supplementary file 1 [file ijms-24-07451-s001.zip › Figures S3-S4-S5.pptx]
